# A Handbook of Midwifery

**Published:** 1898-12

**Authors:** 


**A Handbook of Midwifery.** By W. R. Dakin, M.D. Pp. xx., 629. London: Longmans, Green, and Co. 1897.

Dr. Dakin begins his book with an exceptionally clear account of the processes connected with the development of the ovum. The different steps are simply and clearly described and well illustrated. In the anatomical portion of the work some excellent illustrations show very clearly the relations of the parturient canal, both bony and muscular, and prepare the way for a careful description of the movements performed by the fœtal head in passing through the canal, with the means to be adopted to rectify deviations from normal mechanism, and the way in which by untimely interference this latter can be rendered abnormal. He attaches a due amount of importance to abdominal palpation as a diagnostic method, and shows how this method may by practice be perfected to such an extent as to render vaginal examinations almost unnecessary.

In the description of the methods of rendering the hands aseptic, more dogmatism would be an improvement. In this most important part of the subject too much stress cannot be laid on the minutest details which will affect the perfection of the process, and render their routine employment almost an article of religion for students. A quarter of a minute is certainly too short a time for sterilisation of the hands, especially as usually time is no object, *i.e.*, within limits of say ten minutes; and, moreover, the punctual and careful carrying out of each detail at every examination is absolutely necessary. It would be better to forbid entirely the use of indestructible sponges than to say that their use is inadvisable.

The author gives many valuable hints of a practical nature in his advice as to the conduct of labour and the management of the puerperal state and diseases, but we think errs on the side of over-caution in his advice as to the treatment of puerperal sapræmia: he advises in cases of foul lochia and high temperature to give a vaginal douche and administer ergot for twelve hours, and then, if the symptoms exist, to give an intra-uterine douche. This seems to be rather playing with fire. As the decomposition may not be vaginal, surely the better plan would be to use an intra-uterine douche in all such cases at once and not to allow an interval of twelve hours, during which time the power of resistance will be lessened and microbic virulence increased. The intra-uterine douche is in no respect dangerous if used with circumspection and gentleness; and supposing the contrary were the case, the loss of twelve vital hours in the local treatment of intra-uterine sepsis would be a risk far and away greater. In other respects the book shows signs of great care and considerable original work, and it is a welcome addition to the existing handbooks, among which it will take a prominent place. The illustrations, of which there are four hundred, nearly all original, are excellent, and the index, covering more than forty-one pages, is especially to be commended.

